# Supplementary material for: The mitochondrial fusion protein OPA1 is dispensable in the liver and its absence induces mitohormesis to protect liver from drug-induced injury
Source: Nat Commun. 2023 Oct 23;14:6721. doi: 10.1038/s41467-023-42564-0 (PMC10593833; doi:10.1038/s41467-023-42564-0)
Supplement: Supplementary file 1 — Supplementary Information [file 41467_2023_42564_MOESM1_ESM.pdf]

Suppl. Fig. S1

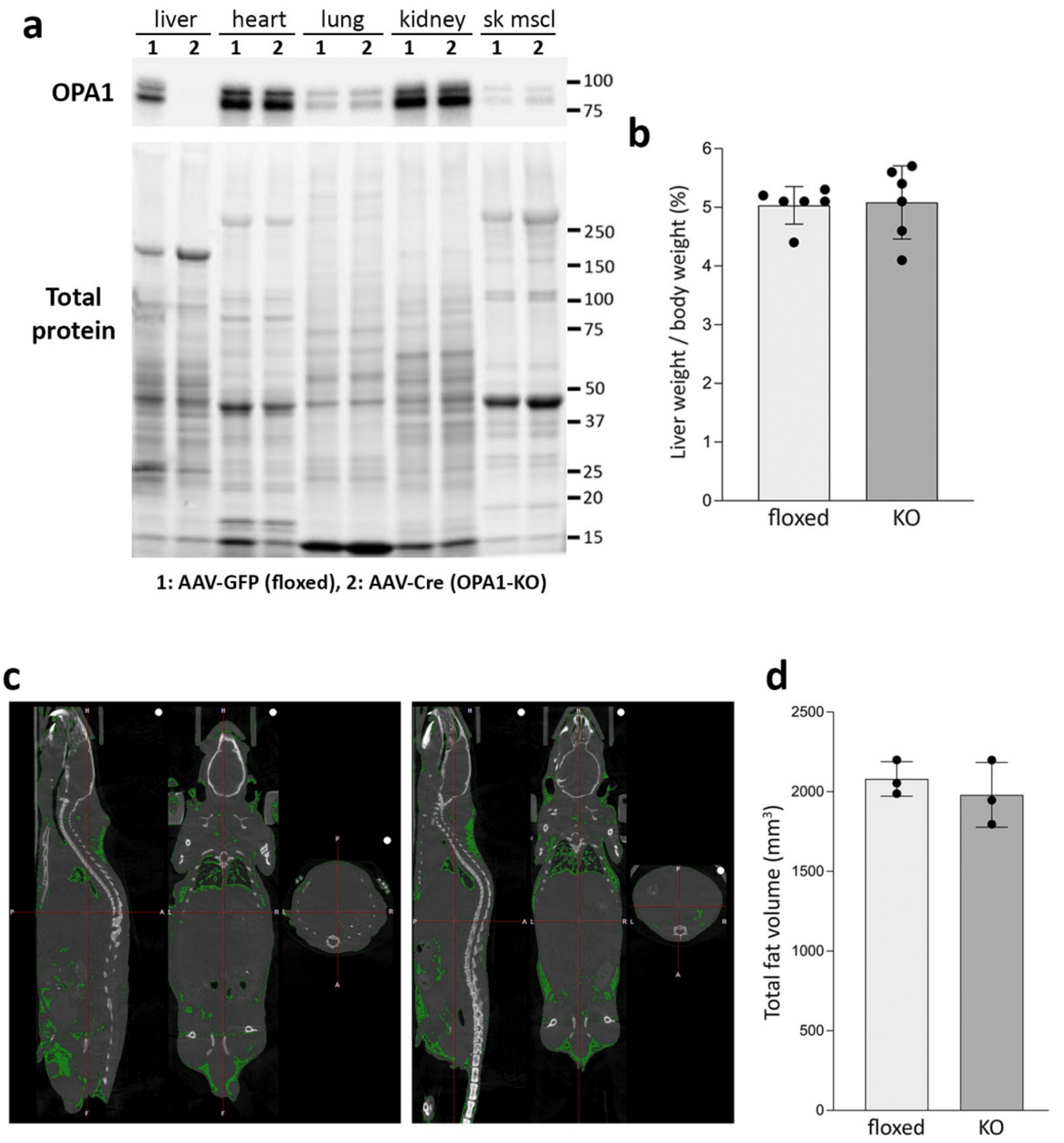

Suppl. Fig. S1. (a) AAV-TBG-Cre injection induces OPA1 KO specifically in the liver. **(b)** There is no difference in liver weight normalized to body weight. n=6 animals per group. Data are presented as mean values +/- SD. Unpaired t test. **(c)** Images of whole-body CT scan of mice. Green color denotes fat area. **(d)** Volume analyses of fat in floxed and OPA1-LKO mice. n=3 animals per group. Data are presented as mean values +/- SD. Unpaired t test. Source data are provided as a Source Data file.

Suppl. Fig. S2

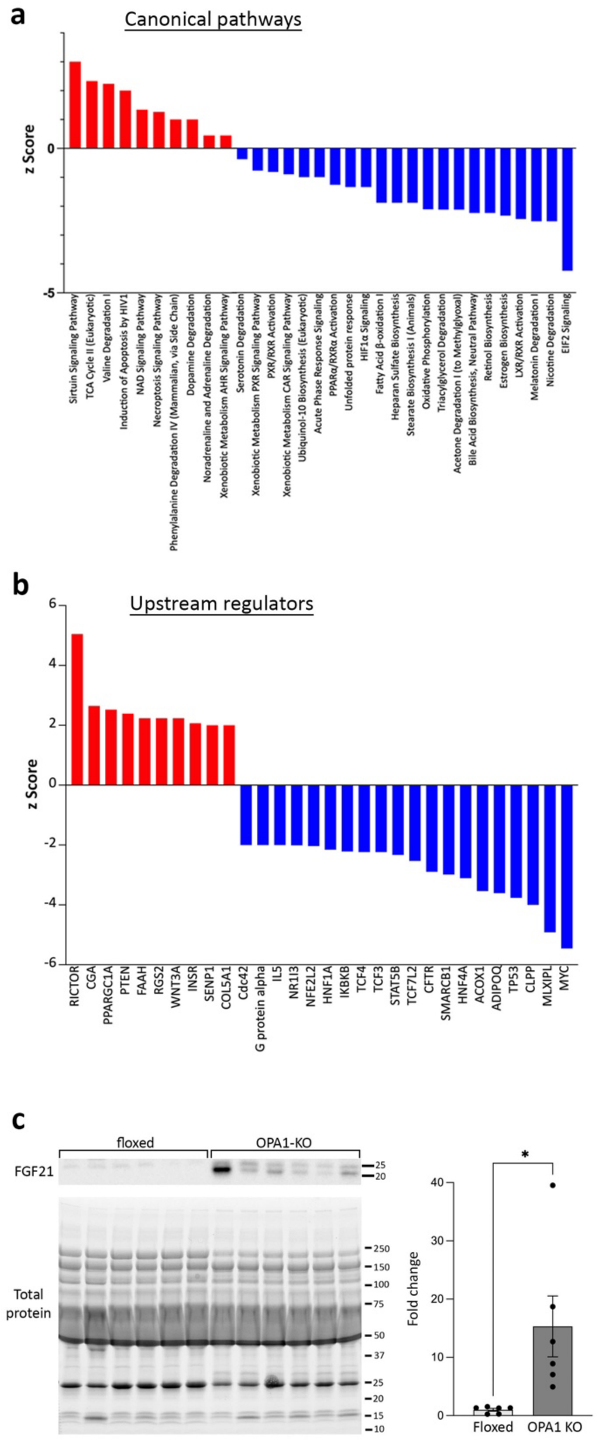

Suppl. Fig. S2. (a, b) IPA analyses of the proteomic data for canonical pathways and upstream regulators. **(c)** An increase of FGF21 in OPA1-LKO mouse serum. n=6 animals per group. Data are presented as mean values +/- SD. Unpaired t test. P=0.0211. Source data are provided as a Source Data file.

Suppl. Fig. S3

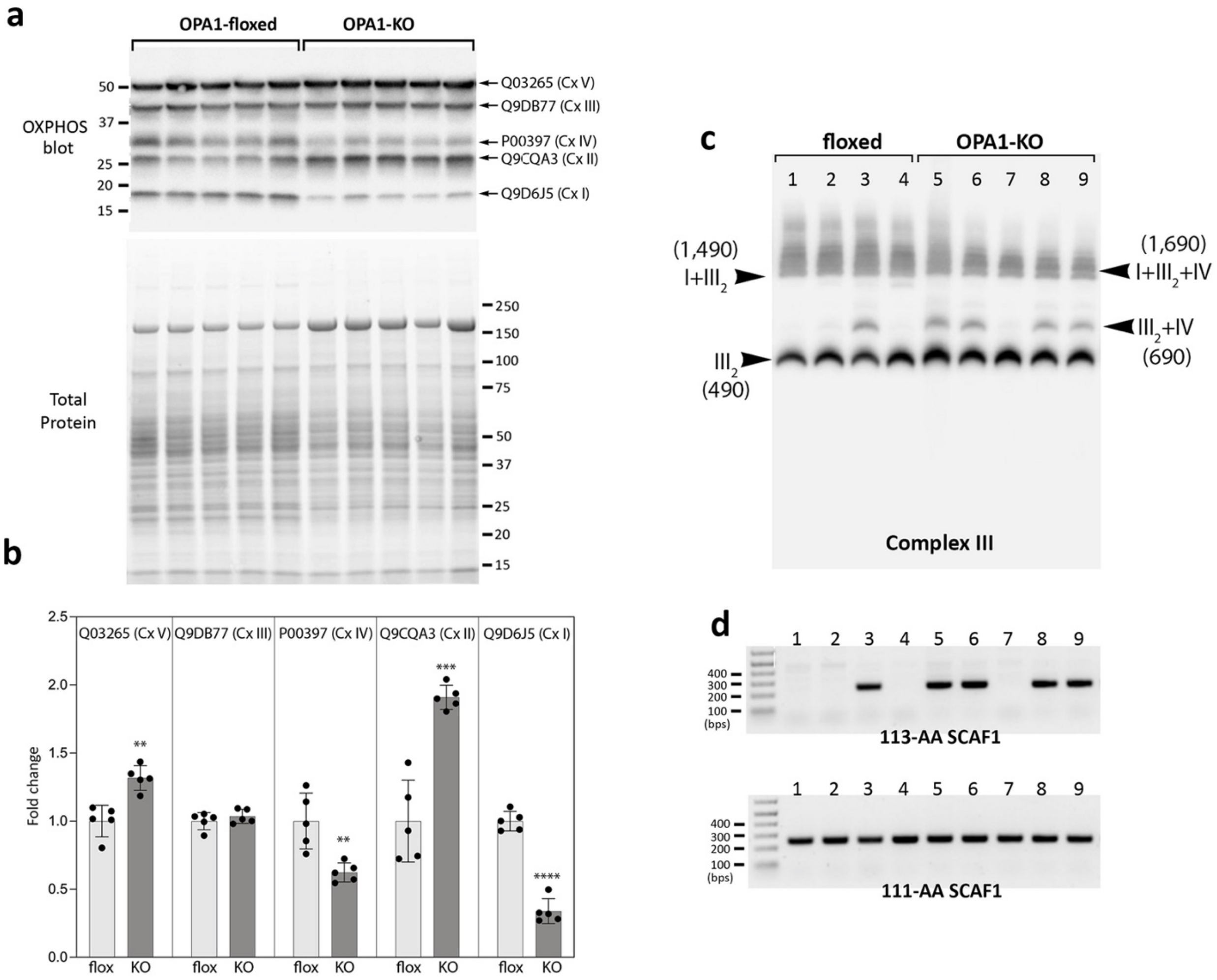

Suppl. Fig. S3. (a) Immunoblot of control and OPA1-KO liver lysates using the OXPHOS complex antibody cocktail. **(b)** Quantification of the immunoblot. n=5 animals per group. Data are presented as mean values +/- SD. Unpaired t test. Cx V: p=0.00131, Cx IV: p=0.0046, Cx II: p=0.0002, Cx I: p<0.0001. **(c)** BNGE for complex III (same blot shown in figure 5d). **(d)** PCR for two SCAF1 isoforms in different mice. Source data are provided as a Source Data file.

Suppl. Fig. S4

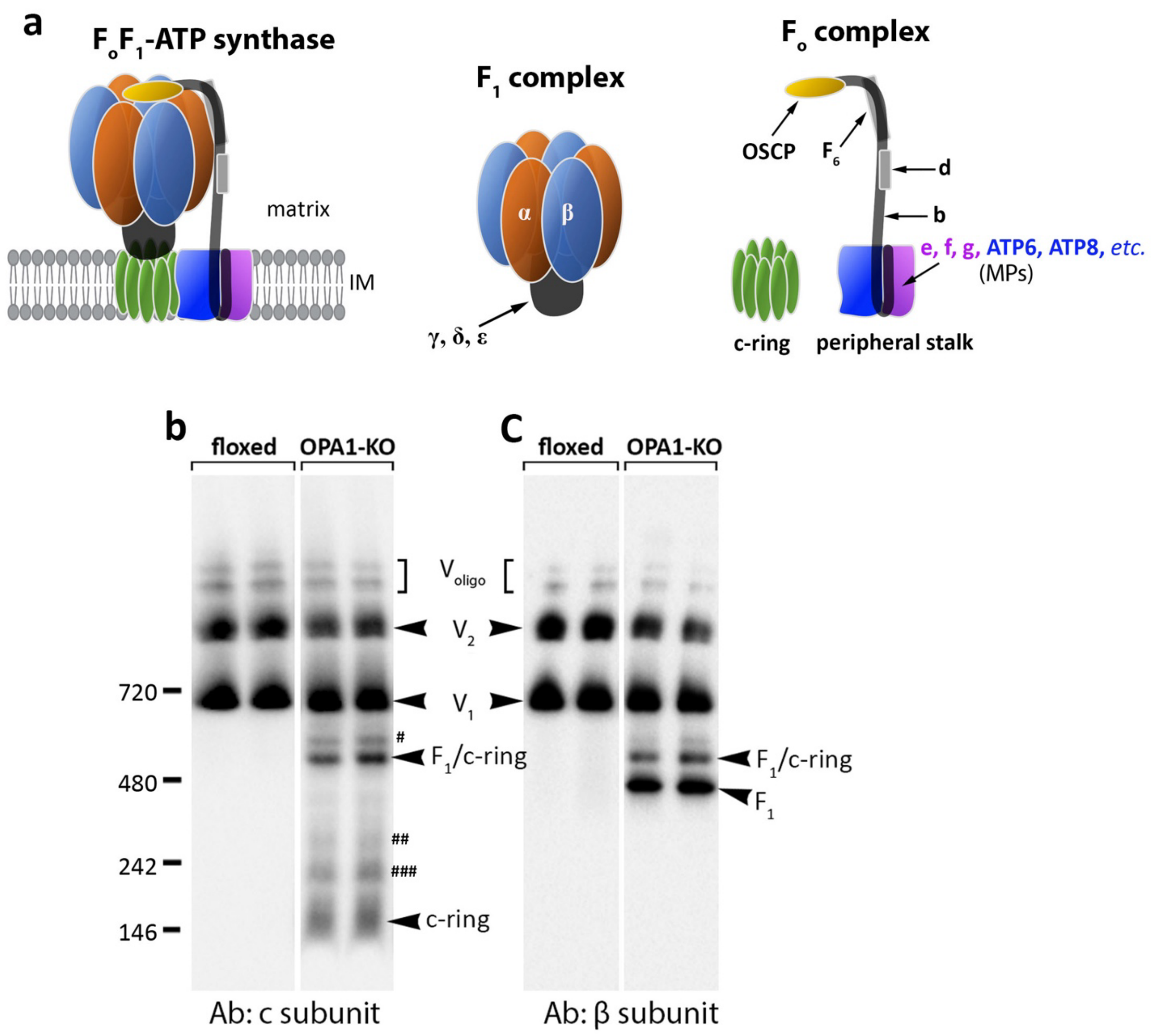

Suppl. Fig. S4. (a) The subunits of complex V (F_oF_1-ATP synthase). The membrane-extrinsic F_1 complex contains α, β, γ, δ, and ε subunits. The F_o complex is membrane-intrinsic and is composed of c-ring and peripheral stalk (b, OSCP, d, and F_6) with multiple membrane proteins (MPs: e, f, g, ATP6, ATP8, etc.). **(b, c)** Subcomplexes accumulated in OPA1 KO mitochondria detected by antibodies against c and β subunits. In addition to c-ring and F1/c-ring, multiple c subunit-containing sub-complexes (#, ##, and ###) are present in OPA1 KO mitochondria.

Suppl. Fig. S5

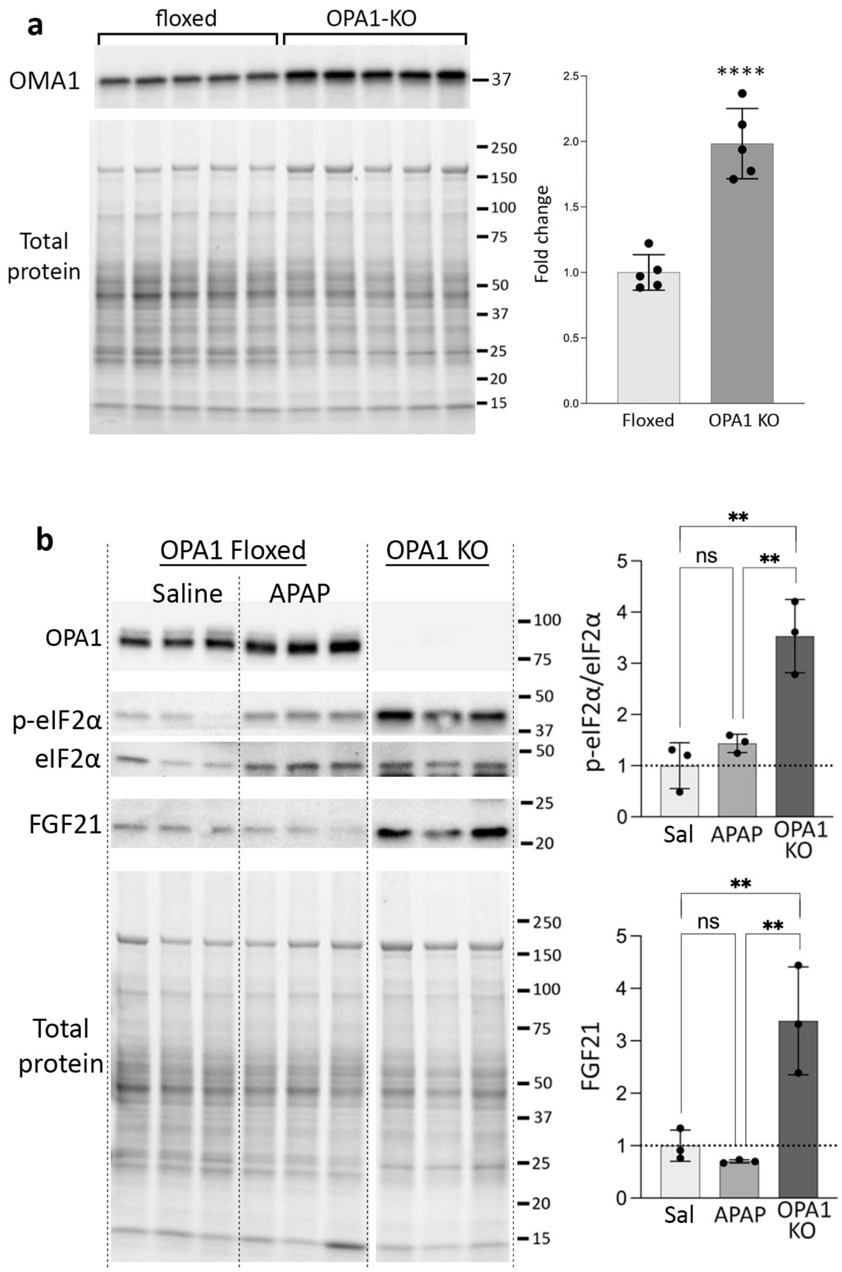

Suppl. Fig. S5. (a) OMA1 immunoblot of floxed and OPA1-KO livers. OPA1 KO significantly increased the OMA1 level. As OMA1 undergoes autocatalytic degradation upon activation, an increase in the OMA1 level indicates its inactivation. n=5 animals per group. Data are presented as mean values +/- SD. Unpaired t test. p < 0.0001. **(b)** APAP treatment does not induce the ISR. Quantification shows no significant change in ISR components in APAP treatment. OPA1-KO livers were included as positive control for ISR. n=3 animals per group. Data are presented as mean values +/- SD. One-way ANOVA with Turkeys multiple comparisons. p=0.0019 and 0.0051 (p-eIF2α); p=0.0078 and 0.0044. Source data are provided as a Source Data file.

Suppl. Fig. S6

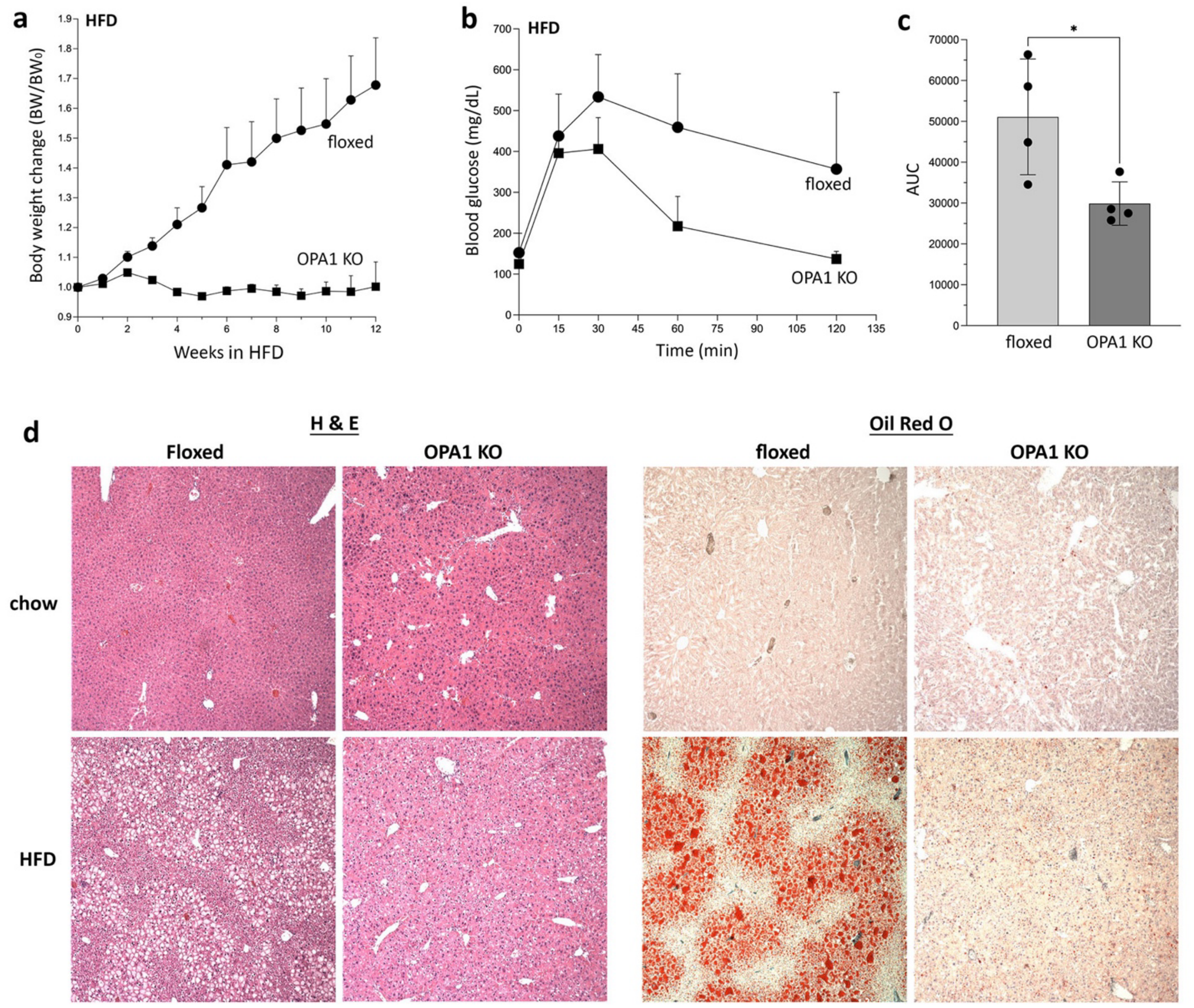

Suppl. Fig. S6. Mitigation of HFD-induced obesity and hepatic steatosis by OPA1 LKO. (a) Body weight change in high fat diet. (b, c) GTT of floxed and OPA1-KO mice after 12-week HFD. n=4 animals per group. Data are presented as mean values +/- SD. Unpaired t test. *, p=0.0310. Source data are provided as a Source Data file. (d) Histology (H&E and Oil red O) showing marked reduction in liver steatosis by OPA1 LKO after 12-week HFD.

Suppl. Fig. S7

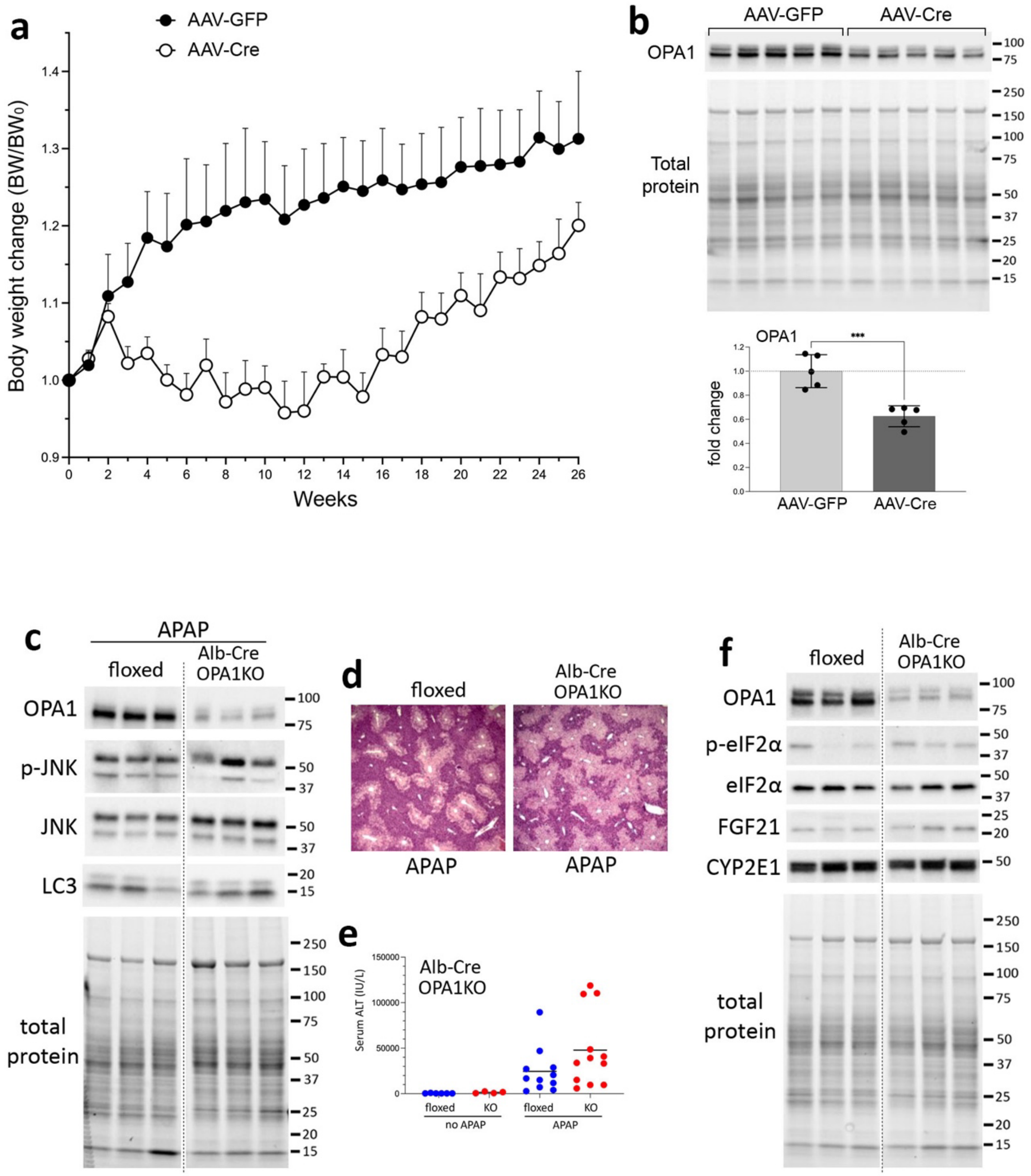

Suppl. Fig. S7. (a) Body weight change in an extended period after AAV-Cre administration. **(b)** OPA1 immunoblot at 24 weeks post-AAV-Cre. n=5 animals per group. Data are presented as mean values +/- SD. Unpaired t test. p=0.0009. **(c – f)** Alb-Cre-mediated OPA1 KO does not protect liver from APAP overdose. **(c)** There are no changes in JNK phosphorylation and LC3-II conversion in Alb-Cre-OPA1 KO compared with floxed controls. **(d)** H&E staining shows marked necrotic areas at 6 hours post APAP in both control and Alb-Cre-OPA1-KO livers. **(e)** Elevated ALT levels in Alb-Cre-OPA1-KO livers, similar to control. **(f)** There are no changes in eIF2α phosphorylation and the levels of FGF21 and CYP2E1 in Alb-Cre-OPA1-KO livers, indicating no ISR induction. Source data are provided as a Source Data file.
