# Supplementary material for: The mitochondrial fusion protein OPA1 is dispensable in the liver and its absence induces mitohormesis to protect liver from drug-induced injury
Source: Nat Commun. 2023 Oct 23;14:6721. doi: 10.1038/s41467-023-42564-0 (PMC10593833; doi:10.1038/s41467-023-42564-0)
Supplement: Supplementary file 3 — Reporting Summary [file 41467_2023_42564_MOESM3_ESM.pdf]

nature portfolio

Corresponding author(s): Yisang Yoon

Last updated by author(s): Oct 4, 2023

Reporting Summary

Nature Portfolio wishes to improve the reproducibility of the work that we publish. This form provides structure for consistency and transparency in reporting. For further information on Nature Portfolio policies, see our Editorial Policies and the Editorial Policy Checklist.

Statistics

For all statistical analyses, confirm that the following items are present in the figure legend, table legend, main text, or Methods section.

n/a	Confirmed	
☐	☒	The exact sample size (*n*) for each experimental group/condition, given as a discrete number and unit of measurement
☐	☒	A statement on whether measurements were taken from distinct samples or whether the same sample was measured repeatedly
☐	☒	The statistical test(s) used AND whether they are one- or two-sided<br>*Only common tests should be described solely by name; describe more complex techniques in the Methods section.*
☐	☒	A description of all covariates tested
☐	☒	A description of any assumptions or corrections, such as tests of normality and adjustment for multiple comparisons
☐	☒	A full description of the statistical parameters including central tendency (e.g. means) or other basic estimates (e.g. regression coefficient) AND variation (e.g. standard deviation) or associated estimates of uncertainty (e.g. confidence intervals)
☐	☒	For null hypothesis testing, the test statistic (e.g. *F*, *t*, *r*) with confidence intervals, effect sizes, degrees of freedom and *P* value noted<br>*Give P values as exact values whenever suitable.*
☒	☐	For Bayesian analysis, information on the choice of priors and Markov chain Monte Carlo settings
☒	☐	For hierarchical and complex designs, identification of the appropriate level for tests and full reporting of outcomes
☒	☐	Estimates of effect sizes (e.g. Cohen's *d*, Pearson's *r*), indicating how they were calculated

Our web collection on statistics for biologists contains articles on many of the points above.

Software and code

Policy information about availability of computer code

Data collection	Immunoblot: Image Lab (v.6.1, Bio-Rad)
Data analysis	Proteomics: Proteome Discoverer version 1.4; R version 4.2.2; Ingenuity pathway Analysis (Qiagen); GraphPad Prism 9<br>Immuniblot quantification: ImageJ (v. 1.53)<br>Statistical analyses: GraphPad Prism (v. 9.5)

For manuscripts utilizing custom algorithms or software that are central to the research but not yet described in published literature, software must be made available to editors and reviewers. We strongly encourage code deposition in a community repository (e.g. GitHub). See the Nature Portfolio guidelines for submitting code & software for further information.

Data

Policy information about availability of data

All manuscripts must include a data availability statement. This statement should provide the following information, where applicable:

- Accession codes, unique identifiers, or web links for publicly available datasets
- A description of any restrictions on data availability
- For clinical datasets or third party data, please ensure that the statement adheres to our policy

The SwissProt mouse database was used for mass spectrometry data search. The mass spectrometry proteomics data have been deposited to the ProteomeXchange Consortium via

the PRIDE partner repository with the dataset identifier PXD040556. All other data supporting the findings of this study are available within the article and its supplementary information files. Source data for some of the figures are provided with the paper.

Human research participants

Policy information about studies involving human research participants and Sex and Gender in Research.

Reporting on sex and gender	N/A
Population characteristics	N/A
Recruitment	N/A
Ethics oversight	N/A

Note that full information on the approval of the study protocol must also be provided in the manuscript.

Field-specific reporting

Please select the one below that is the best fit for your research. If you are not sure, read the appropriate sections before making your selection.

- [x] Life sciences
- [ ] Behavioural & social sciences
- [ ] Ecological, evolutionary & environmental sciences

For a reference copy of the document with all sections, see nature.com/documents/nr-reporting-summary-flat.pdf

Life sciences study design

All studies must disclose on these points even when the disclosure is negative.

Sample size	Sample size of each experiment is indicated in the figure legend and presented as the individual data points. No sample size calculation was performed because experimental repetitions showed high degree of consistency, which allows low n in some datasets for assessing statistical significance.
Data exclusions	We found that the mouse body weight is the bona fide predictor of whether the KO of OPA1 is complete or not following the AAV-Cre adminstration. Therefore, we excluded mice that gained weight continuously after AAV-Cre administration. These mice had incomplete KO, expressing >25% of the wild type level of OPA1 with no ISR induction.
Replication	We performed more than 10 sets of AAV injection, in which mouse numbers in each set range from 4 to 11 for both AAV-GFP and AAV-Cre. For any given experiments, data collected were consistent and reproducible among experimental sets.
Randomization	Mice were randomly assigned for AAV injection, and APAP treatment.
Blinding	Samples were blinded for mass spec analyses and histology. Other experiments were not blinded to allow paired assays for control and KO samples and also to allow specific sample arrangements in gels.

Reporting for specific materials, systems and methods

We require information from authors about some types of materials, experimental systems and methods used in many studies. Here, indicate whether each material, system or method listed is relevant to your study. If you are not sure if a list item applies to your research, read the appropriate section before selecting a response.

Materials & experimental systems

n/a	Involved in the study	
[ ]	[x]	Antibodies
[ ]	[x]	Eukaryotic cell lines
[x]	[ ]	Palaeontology and archaeology
[ ]	[x]	Animals and other organisms
[x]	[ ]	Clinical data
[x]	[ ]	Dual use research of concern

Methods

n/a	Involved in the study	
[x]	[ ]	ChIP-seq
[x]	[ ]	Flow cytometry
[x]	[ ]	MRI-based neuroimaging

Antibodies

Antibodies used	Primary antibodies against:<br>OPA1: BD Biosciences, 612606; 1:1000<br>Caspase-3: Cell Signaling Tech, 9662; 1:1000<br>PARP-1: Cell Signaling Tech, 9542; 1:1000)<br>beta-actin: Sigma, A1978; 1:40000<br>TOM20: Proteintech, 11802-1-AP; 1:1000<br>Cytochrome c: BD Biosciences, 556432; 1:5000<br>eIF2alpha: Cell Signaling Tech, 9722; 1:1000)<br>Phospho-eIF2alpha: Cell Signaling, 9721; 1:1000<br>FGF21: Proteintech, 26272-1-AP; 1:1000<br>LC3 A/B: Cell Signaling Tech, 4108; 1:1000<br>PGC1alpha: Invitrogen, PA5-38022; 1:500<br>OMA1: Santa Cruz Biotechnology, sc-515788; 1:100<br>Mitochondria total OXPHOS rodent WB cocktail: Abcam, ab110413; 1:1000<br>JNK: Cell Signaling Tech, 9252; 1:1000<br>p-JNK-Thr183/Tyr185: Cell Signaling Tech, 9255; 1:500<br>MCU: Sigma, HPA016480; 1:1000<br>NCLX: Proteintech, 21430-1-AP; 1:1000<br>CypD: Proteintech, 18466-1-AP; 1:1000<br>MnSOD: BD Biosciences, 611580; 1:1000<br>GPx1/2: Santa Cruz Biotechnology, sc-133160; 1:200<br>CYP2E1: Proteintech, 19937-1-AP; 1:300<br>NDUFA5 (CxI): GeneTex, GTX111016; 1:500<br>Succinate dehydrogenase subunit B (CxII): GeneTex, GTX113833; 1:500)<br>UQCRC2 (CxIII): GeneTex, GTX114873; 1:1000<br>COX4 (CxIV): GeneTex, GTX114330; 1:1000<br>ATP5G1/G2/G3 (CxV c subunits): Abcam, ab180149; 1:1000<br>ATP synthase subunit beta: Molecular Probes, A-21351; 1:500<br><br>Secondary antibodies:<br>HRP-conjugated goat anti-Rabbit IgG (H+L): Jackson ImmunoResearch, 111-035-003, 1:5,000 - 1:10,000<br>HRP-conjugated goat anti mouse IgG (H+L): Jackson ImmunoResearch, 115-035-003, 1:5,000 - 1:10,000
Validation	The suppliers have validated their antibodies, as described in their websites, and they recognized bands at their expected molecular weights. All antibodies have been cited by other publications.<br>Caspase-3 Cell Signaling Tech, 9662: validated by KO cells by the supplier<br>PARP-1 Cell Signaling Tech, 9542: validated by cell death inducer by the supplier<br>Phospho-eIF2alpha and eIF2alpha: Cell Signaling Tech, 9721 and 9722: validated by stress inducers by the supplier<br>LC3 A/B: Cell Signaling Tech, 4108: validated by autophagy inducer by the supplier<br>p-JNK-Thr183/Tyr185 and JNK: Cell Signaling Tech, 9255 and 9252: validated by cell death inducer by the supplier<br>MCU: Sigma, HPA016480: validated by siRNA by the supplier<br>NCLX: Proteintech, 21430-1-AP: validated by siRNA (PMID: 26089852)<br>CYP2E1: Proteintech, 19937-1-AP: validated by siRNA (PMID: 34758665)

Eukaryotic cell lines

Policy information about cell lines and Sex and Gender in Research

Cell line source(s)	OPA1-null MEFs: ATCC CRL2995<br>WT and Mfn-DKO MEFs: from David Chan (CalTech)
Authentication	OPA1-null MEFs - PMID: 17709429 PMCID: PMC2064540 DOI: 10.1083/jcb.200704110<br>WT and Mfn-DKO MEFs - PMID: 15899901 DOI: 10.1074/jbc.M503062200
Mycoplasma contamination	No known mycoplasma contamination by ATCC test
Commonly misidentified lines<br>(See ICLAC register)	N/A

Animals and other research organisms

Policy information about studies involving animals; ARRIVE guidelines recommended for reporting animal research, and Sex and Gender in Research

Laboratory animals	Male OPA1-fl/fl mice (C57BL/6J) were used in this study. Eight week-old mice were used for AAV administration and further kept for 8-12 weeks until the experiments. Alb-Cre mice were obtained from Jackson Laboratory. Mice were kept under controlled temperature and lighting (20–22C; 12-h dark-light period) in 50% humidity and with free access to a standard chow diet and water, except for the experiments requiring fasting.
Wild animals	No wild animals were used in this study

Reporting on sex	Only male mice were used in this study based on the literature showing insufficient AAV transduction in the liver of female mice, which does not allow complete KO necessary for this study. The APAP model also shows the inconsistency in causing liver injury in female mice by APAP overdose.
Field-collected samples	The study did not involve field-collected samples.
Ethics oversight	All animal experiments were performed according to the NIH guideline for the care and use of laboratory animals and the protocols approved by the IACUC at Augusta University (IACUC Protocol # 2016-0801).

Note that full information on the approval of the study protocol must also be provided in the manuscript.
